# Retraction: MicroRNA-223 decreases cell proliferation, migration, invasion and enhances cell apoptosis in childhood acute lymphoblastic leukemia via targeting Forkhead box O1

**DOI:** 10.1042/BSR-2020-0485_RET

**Published:** 2024-05-29

**Keywords:** acute lymphoblastic leukemia, cell apoptosis, cell proliferation, migration and invasion, miR-223, FOXO1

This article is being retracted from *Bioscience Reports* at the request of the Editor-in-Chief and the Editorial Board following receipt of a notification from a reader, alerting the Editorial Board to multiple concerns surrounding the data presented in this paper. Specifically, concerns regarding:

- Figure 2C CCRF-CEM NC-mimic panel and CCRF-CEM miR-223-inhibitor panel parts of which seem to appear as Figure 1B MCF-7 10 µM and Figure 1B MDA-MB-231 0 µM respectively, from Shen et al, 2020 (DOI: 10.2147/cmar.s246691) [retracted]
- Figure 2D miR-223 mimic panel, Figure 5D si-NC+NC-inhibitor panel, and Figure 5D si-FOXO1+miR-223 inhibitor panel which share unexpected similarities with Figure 4A CCRF-CEM sh-GAS2 panel, Figure 4A Jurkat GAS2 panel, and Figure 4A CCRF-CEM NC panel respectively, from Kong et al, 2020 (DOI: 10.2147/ott.s236854)
- Parts of Figure 2D NC-mimic panel which seem to appear in Figure 2D NC-inhibitor panel
- The veracity of data presented in Figure 4C
- Figure 4F α-tubulin panel which seems to appear in Figure 3A GAPDH panel from Shen et al, 2020 (DOI: 10.2147/CMAR.S246691) [retracted]
- Figure 5A α-tubulin panel which seems to appear as Figure 4B Jurkat β-actin panel from Kong et al, 2020 (DOI: 10.2147/ott.s236854)
- Figure 5A FOXO1 panel a part of which appears as Figure 3B YAP panel from Shen et al, 2020 (DOI: 10.2147/cmar.s246691) [retracted]
- Figure 5C NALLM-6 si-NC+NC-inhibitor panel and NALLM-6 si-FOXO1+miR-223 inhibitor panel parts of which seem to appear as Figure 2D Mock panel and pcDNA3.1-NC panel respectively, from Shao et al, 2020 (DOI: 10.2147/ott.s243736)
- Figure 5E Migration si-NC+NC-inhibitor panel a part of which appears in Figure 5E Migration si-FOXO1+miR-223 inhibitor panel
- Figure 5F si-FOXO1+miR-223 inhibitor panel a part of which appears in Figure 3B NC-inhibitor panel in this paper, Figure 3B miR-223 inhibitor panel in this paper, and Figure 2D SK-Hep-1 LD panel from Shi et al, 2020 (DOI: 10.2147/ott.s248797)

The authors have been contacted with regards to the retraction and have not responded to the Journal's queries or the concerns raised. Given the extent of the issues raised, the Editorial Board stand by the decision to retract the article.

